# Mechanical properties and failure behaviors of the interface of hybrid graphene/hexagonal boron nitride sheets

**DOI:** 10.1038/srep31499

**Published:** 2016-08-16

**Authors:** Ning Ding, Xiangfeng Chen, Chi-Man Lawrence Wu

**Affiliations:** 1 Department of Physics and Materials Science, City University of Hong Kong, Hong Kong SAR, PR China; 2 Shandong Academy of Sciences, Jinan, PR China

## Abstract

Hybrid graphene/*h*-BN sheet has been fabricated recently and verified to possess unusual physical properties. During the growth process, defects such as vacancies are unavoidably present at the interface between graphene and *h*-BN. In the present work, typical vacancy defects, which were located at the interface between graphene and *h*-BN, were studied by density functional theory. The interface structure, mechanical and electronic properties, and failure behavior of the hybrid graphene/*h*-BN sheet were investigated and compared. The results showed that the formation energy of the defective graphene/*h*-BN interface basically increased with increasing inflection angles. However, Young’s modulus for all graphene/*h*-BN systems studied decreased with the increase in inflection angles. The intrinsic strength of the hybrid graphene/*h*-BN sheets was affected not only by the inflection angles, but also by the type of interface connection and the type of defects. The energy band structure of the hybrid interface could be tuned by applying mechanical strain to the systems. These results demonstrated that vacancies introduced significant effects on the mechanical and electronic properties of the hybrid graphene/*h*-BN sheet.

The mechanical performance of graphene and graphene-like materials is one of the most important aspects for their assembling and application in electronic, optical and thermal nano-devices^1^,^2^,^3^. As reported in previous works^4^,^5^,^6^,^7^,^8^,^9^,^10^, defects such as vacancies and grain boundaries could introduce significant influence on the mechanical properties of low-dimensional materials. Recently, hybrid graphene/hexagonal boron nitride (*h*-BN) lateral heterostructure has been fabricated using chemical vapor deposition (CVD) method^11^,^12^,^13^. For instance, in 2012, Levendorf and co-workers prepared a type of hybrid graphene/*h*-BN sheet by a “patterned re-growth” method which allows for the spatially controlled synthesis of lateral junctions between graphene and *h*-BN^12^. The hybrid graphene/*h*-BN is of particular interest because it possesses unusual physical properties, such as magnetism^14^, unique thermal transports^15^ and robust half-metallic behavior^16^,^17^. Thus, it has great potential application in the development of future nano-devices^18^,^19^,^20^,^21^. In addition, it has been proved that the shape of the interface between graphene and *h*-BN domains on the hybrid graphene/*h*-BN sheet affects its electronic properties significantly^17^,^22^,^23^,^24^,^25^.

More recently, using scanning tunneling microscopy (STM), Loh and co-workers clearly observed discontinues and dislocations along the interface of a graphene/*h*-BN surface produced via a two-step sequential CVD method^26^. Due to the results of the lattice mismatch between graphene and *h*-BN, interfacial strain and uneven growth at the interface may exist. Discontinues, such as vacancies, fault lines and cracks, are unavoidably present near and along the graphene/*h*-BN interface to reduce elastic strain energy. As we know, the atoms in the defect are not in perfect crystalline arrangement. The mechanical properties of this hybrid material may be further affected and may give rise to large impact on the performance of devices based on such material. Vacancies are one of the most popular point defects on two-dimensional (2D) material surface. Similar to graphene and some other graphene-like materials^27^,^28^,^29^, vacancies at the graphene/*h*-BN interface should serve as fracture nucleation center under high tension load. Their mechanical properties are more complicated due to the existence of two different types of atomic lattices. It is therefore worthwhile to study the possible relationship between the mechanical properties of graphene/*h*-BN and the defect parameters. However, recent experimental and theoretical studies focused on the patterns, electronic and thermoelectric properties of the graphene/*h*-BN interface^30^,^31^,^32^,^33^. To the best of our knowledge, no systematic researches were performed to investigate the mechanical properties of the graphene/*h*-BN interface.

The present work aims to investigate the potential effect of the interface on the mechanical properties and failure behavior of the hybrid graphene/*h*-BN sheet. In particular, the perfect interface and interfaces with typical vacancy defects located along the interface were studied by density functional theory (DFT). The interface structure, defect formation energy, mechanical properties, changes of the energy band during tensile process, and failure behavior of the hybrid graphene/*h*-BN interface with and without defects were studied and compared.

## Computational details and models

### Computational details

The mechanical properties and failure behavior of the interface of hybrid graphene/*h*-BN sheet was investigated using DFT method. Generalized gradient approximation (GGA) with Perdew-Burke-Ernzerhof (PBE) function was chosen to describe the graphene/*h*-BN interface^34^. The calculation was based on double-numeric quality with a polarization functions (DNP) basis set. The periodic boundary condition was performed on the computational unit cell. For all models studied, a vacuum with a height of 20 Å was placed above the graphene/*h*-BN surface to minimize the influence between adjacent layers. In addition, the distance between adjacent interfaces was set as more than 20 Å to minimize the influence between them. Å 10 × 10 × 1 k-point mesh was set up during the geometry equilibrium with all the atomic structure parameters fully relaxed. All calculations were realized using the DMol3 module (Accelrys Inc.).

### Models of the interfaces

**Figure 1:**
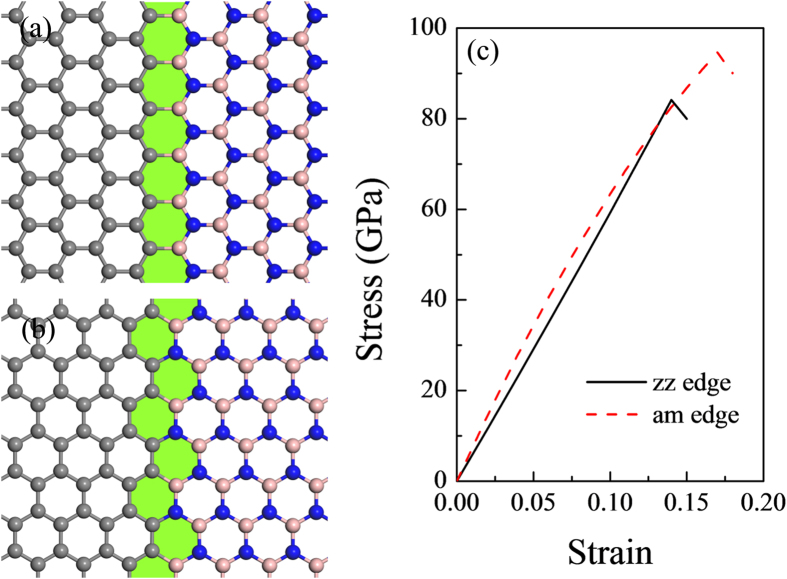
Atomic structures of the hybrid graphene/*h*-BN interface with (**a**) a zigzag edge and (**b**) an armchair edge; (**c**) their corresponding stress-strain curves.

As reported by previous experimental observations^13^, joining of graphene and *h*-BN domains could form an interface with either a zigzag linking edge or an armchair linking edge. In the present work, both the zigzag edge interface (G-BN-zz) and armchair edge interface (G-BN-am) between graphene and *h*-BN domains were considered (as shown in Fig. 1). It is necessary to note that due to the periodic conditions added to both the *x* and *y* directions of the computing supercell, the G-BN-zz system contains two types of zigzag edges. That is, while one of the interface edges is a carbon-boron (C-B) zigzag shape, its adjacent interface edge possesses a carbon-nitrogen (C-N) configuration.

**Figure 2:**
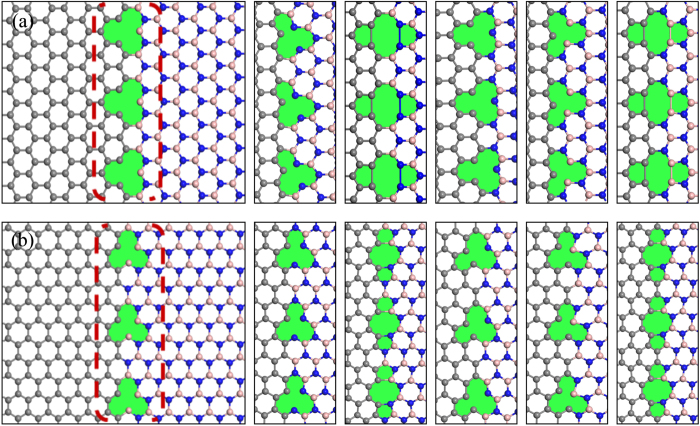
Atomic structures and nomenclature of the vacancy defects at the graphene/*h*-BN interface with (**a**) a zizzag edge (from left to right: zz-SV(CB)-C, zz-SV(CB)-B, zz-DV(CB)-CB, zz-SV(CN)-C, zz-SV(CN)-N, zz-DV(CN)-CN) and (**b**) an armchair edge (from left to right: am-SV-N, am-SV-B, am-DV-BN, am-SV-C(N), am-SV-C(B), am-DV-CC).

For either the zigzag interface or the armchair interface, six typical vacancy defects located at the interface were selected as probes to analyze the effects of vacancies on the mechanical properties and failure behavior of the interfaces. Due to the combinations of single atom vacancies (SV) or double atom vacancies (DV), the interface may turn into different line defects. The nomenclature and structure of the defect models are shown in Fig. 2. As an illustration on the nomenclature used in this work, a defect line formed by SV defect with a carbon atom lost at an zigzag edge interface is named as zz-SV(CB)-C (at the C-B edge) or zz-SV(CN)-C (at the C-N edge). Also, a defect line at the armchair edge interface, which was formed by the single carbon vacancies, is expressed as am-SV-C(N) (the lost carbon atom was connected with a nitrogen atom) or am-SV-C(B) (the lost carbon atom was connected with a boron atom). It was necessary to note that the labels “am (zz)” here denoted the armchair (zigzag) shape of the interface edge rather than the armchair (zigzag) direction. Actually, the tensile load added to an am-** system in the present work was along the zigzag direction of the surface, and vice versa.

## Results and Discussion

### Structures of the graphene/*h*-BN interfaces

As shown in Figure S1, the graphene/*h*-BN sheets with defects usually exhibit a slight warping. To reflect the structural changes of graphene/*h*-BN interface due to the vacancy defects, inflection angle was defined (see Figure S1) and collected in Table 1. The inflection angles of the hybrid graphene/*h*-BN systems ranged from 3° to 35°. Generally speaking, the existence of vacancies enhanced the warpage of the hybrid graphene/*h*-BN sheet to keep the system stable. As shown in Table S1, structural parameters around the vacancies, such as bond lengths and bond angles, showed corresponding changes. Comparing to the pristine graphene and *h*-BN, less than 1% changes for the C-C-C angles, B-N-B (or N-B-N) angles, C-C bonds and B-N bonds were observed on the hybrid graphene/*h*-BN interface without defects (for both the zigzag and armchair edge interface).

For the C-B zigzag interface with vacancies, the maximum elongation (3.8%) of C-B bond relative to the system without defects occurred at the vacancy of the zz-SV(CB)-B system; while for the C-N zigzag interface with vacancies, the maximum change (2.8%) of C-N bond was found at the interface of zz-SV(CN)-N system. When comparing the system with defects and an armchair interface without defects, the changes of C-B and C-N bonds were up to 13.3% and 2.9%, respectively. The maximum elongation of the C-B bond appeared at the interface of the am-SV-B system; while that of the C-N bond was found at the interface of the am-SV-C(B) system. Due to the dangling bond around the vacancies, several atoms (N or B) around the vacancies exhibited a slight elevation (0.35 Å to 0.58 Å) from the graphene/*h*-BN surface.

### Formation energies of the graphene/*h*-BN interfaces

Formation energy was introduced to describe the thermodynamic stability of the vacancies on the hybrid graphene/*h*-BN interface. It can be expressed as^4^,^5^


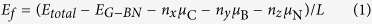


where *E*_*total*_ and *E*_*G−BN*_ are the energies of the hybrid graphene/*h*-BN sheet with and without vacancies, respectively. *μ*_C_, *μ*_B_ and *μ*_N_ denote the increased (*n* > 0) or decreased (*n* < 0) chemical potential of carbon, boron and nitrogen atoms, respectively. *L* is the periodic length along the interface line. Formation energies of all the graphene/*h*-BN systems are shown in Table 1. It can be seen that the values of formation energies for all of the graphene/*h*-BN interfaces with defects ranged from 6.3 eV to 14.4 eV, which were comparable to that of the defect on pristine graphene and *h*-BN sheet^4^,^5^.

**Figure 3:**
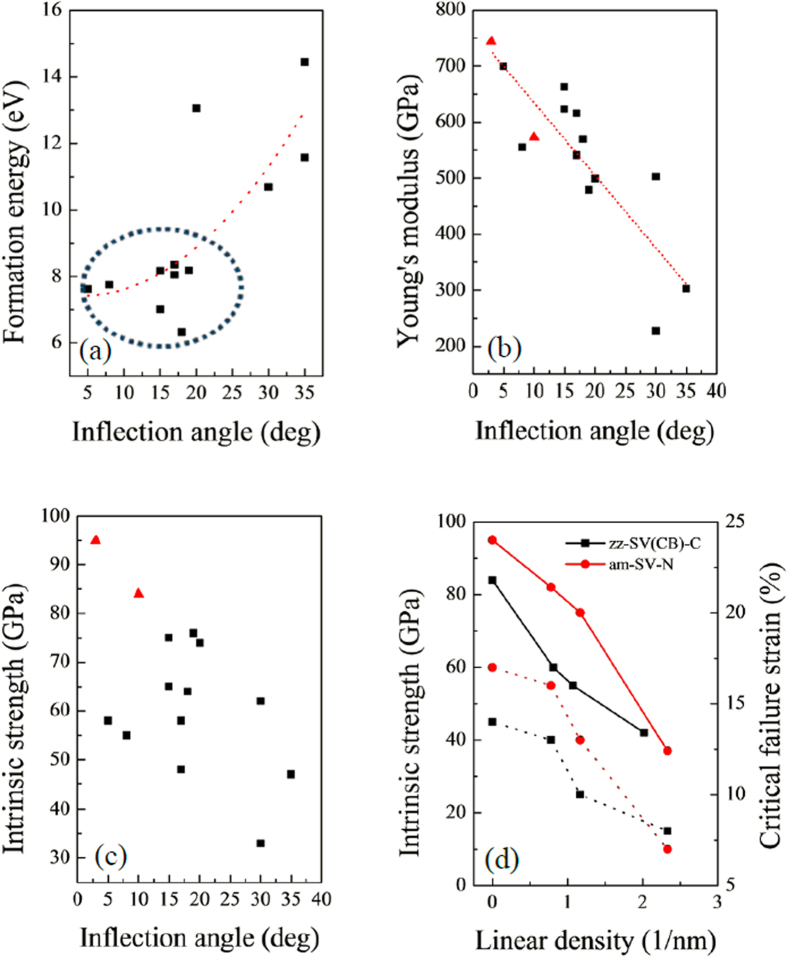
(**a**) Formation energy, (**b**) Young’s modulus and (**c**) intrinsic strength of the graphene/*h*-BN interfaces as functions of the inflection angles. The red triangles denote the defect-free graphene/*h*-BN interface. (**d**) Left-hand scale: the intrinsic strength as a function of linear density of the defect unit along the interface (solid-line); Right-hand scale: the critical failure strain as a function of linear density of the defect unit along the interface (dot-line).

As shown in Fig. 3(a), the formation energy of the defective graphene/*h*-BN interface basically increased with increasing inflection angles. Actually, graphene/*h*-BN interfaces with SV defects exhibited relatively lower formation energies (6.3 eV to 8.4 eV; see the blue circle in Fig. 3(a)) than the interface with DV defects (10.7 eV to 14.4 eV). During the formation of the DV defect at the interface, more energy was needed to compensate such defect induced structural modification than that of the SV defect. In addition, a DV defect usually makes the surface bend more to keep the surface stable.

### Mechanical properties of the graphene/*h*-BN interfaces

**Figure 4:**
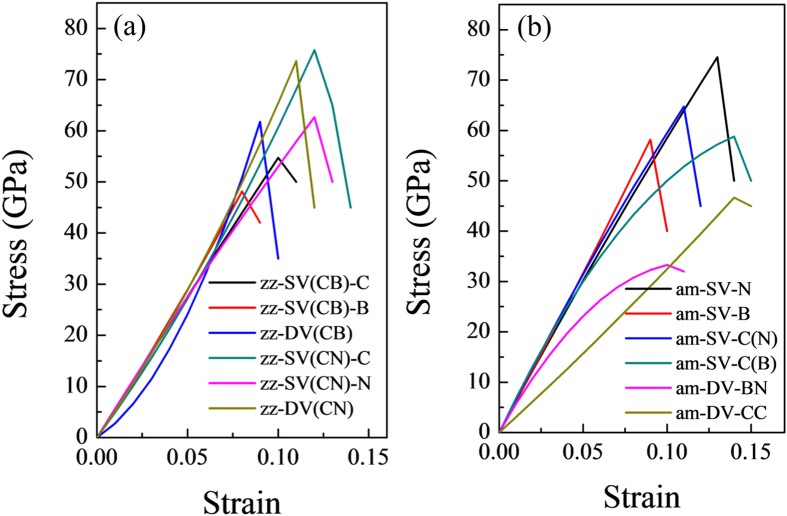
Stress-Strain curves of the defective graphene/*h*-BN system with (**a**) a zigzag edge and (**b**) an armchair edge.

To compare the influence of defects on the mechanical properties of graphene/*h*-BN interface, a series of uniaxial tensile test along the tangential direction of the graphene/*h*-BN surface and perpendicular to the interface were performed. Stress-strain curves of graphene/*h*-BN interface with (see Fig. 1(c)) and without defects (see Fig. 4) were plotted, based on the relationship between strain energy and stress.

### Young’s modulus of the graphene/*h*-BN interfaces

Young’s modulus defines the relationship between stress and strain. It marks the rigidity of materials, *i.e.* the higher the Young’s modulus, the harder to deform the material. In this work, the Young’s modulus of graphene/*h*-BN was calculated as 573 GPa and 744 GPa for the zigzag edge interface and armchair edge interface, respectively. When compared with the pristine graphene and *h*-BN, the Young’s modulus of the zigzag edge interface was reduced to 55.5% (57.5%) of that for graphene (*h*-BN), while that of the armchair edge interface reduced to 72.0% (86.2%) of that for graphene (*h*-BN) (see Table 1). Thus, the graphene/*h*-BN interface even without defects led to a significant decrease in the elastic property of the 2D surface. For the models with defects, the Young’s modulus ranged from 570 GPa~228 GPa for the systems with a zigzag edge and 700 GPa~303 GPa for the systems with an armchair edge, respectively. In general, the Young’s modulus of the models with defects was lower than that of their respective interface without defects, which fully illustrate the effect of the damage of vacancies on the graphene/*h*-BN interface.

As shown in Fig. 3(b), Young’s modulus of all graphene/*h*-BN systems basically decreased with increasing inflection angle. The relatively flat interface, such as BN-G-am (744 GPa) and am-SV-C(B) (700 GPa) systems, has the highest Young’s modulus amongst all systems. When the inflection angle of the interface reaches more than 20°, Young’s modulus of the systems significantly decreases to less than 70% of that of the graphene/*h*-BN interface without defects. The fitted line in Fig. 3(b) shows that the relationship between Young’s modulus and inflection angles can be expressed as


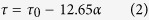


where *τ*_0_ = 761 GPa is the approximate highest Young’s modulus of the graphene/*h*-BN interface without defects. Obviously, the warpage of graphene/*h*-BN interface significantly decreases the Young’s modulus of this hybrid material. As we know, a curved surface is usually easier to be deformed than a completely flat surface, as the initial deformation for a curved surface was the spreading of the surface rather than the elongation of the chemical bonds. Thus, the higher the inflection angle, the easier the deformation to occur at the graphene/*h*-BN interface.

### Intrinsic strengths of the graphene/*h*-BN interfaces

The morphology of stress-strain curves for both the graphene/*h*-BN interface with and without defects show obvious brittle fracture characteristic (see Figs 1(c) and 4), *i.e.*, rupture occurs without any prior noticeable change in the rate of elongation. Intrinsic strength, which was defined as the highest stress point along the stress-strain curve, was obtained to evaluate the mechanical performance of graphene/*h*-BN interface. The intrinsic strength of graphene/*h*-BN zigzag interface without defects was 84 GPa. It was about 80% of that of the prinstine graphene (along armchair direction) and nearly equal to that of the prinstine *h*-BN (along armchair direction). The graphene/*h*-BN armchair interface possessed an intrinsic strength of 95 GPa, which was 13% higher than that of the zigzag interface.

As reported in previous works^4^,^5^, the intrinsic strength of graphene (or *h*-BN) sheet with grain boundaries on the surface showed an obvious linear relationship with the inflection angles of the surface, i.e., the intrinsic strength basically showed linear decrease with the increase of the inflection angles. As shown in Fig. 3(c), for the hybrid graphene/*h*-BN sheet, although the increase of the inflection angles affect the intrinsic strength of the defective systems to a certain extent, no clear linear relationship was found between the intrinsic strength and the inflection angles. The warpage of the graphene/*h*-BN sheet might decrease the strength of the surface as a larger inflection angle effectively causes a 2D surface to become more bulk, which is close to its 3D structures. Nevertheless, apart from the strength decrease, other factors may also affect the strength of the system.

**Figure 5:**
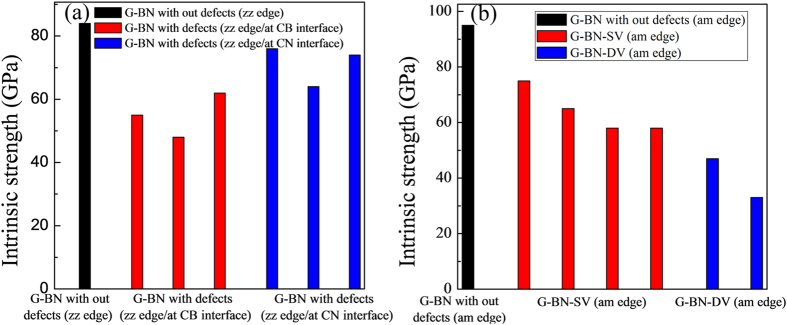
Intrinsic strength of the hybrid graphene/h-BN interface with (**a**) a zigzag edge and (**b**) an armchair edge.

After a careful analysis of the results, it was found that for the models with defects and a zigzag edge interface, the intrinsic strength showed close relationship with the type of the interface (C-B edge or C-N edge) where the vacancies were located at. Vacancies which were located at the C-B edge introduced more detrimental effect on the intrinsic strength of the systems than that located at the C-N edge (shown in Fig. 5(a)). As the interaction energy between carbon and nitrogen atoms are much higher than that between carbon and boron atoms, the C-N edge interface possesses higher strength than that of the C-B edge interface. The existence of vacancies on the C-B edge interface made this originally weak interface to have a lower intrinsic strength. As for the models with defects and an armchair edge interface, the systems with DV defects showed lower intrinsic strength than that of the systems with SV defects. As shown in Fig. 2, every DV defect on the armchair edge interface formed a relatively long and narrow defect unit along the interface just like a nano-crack which is perpendicular to the tensile load. Their shapes were different to the DV defects on the zigzag edge interface. Such structure brought more detrimental effect on the intrinsic strength of the armchair edge interface than the SV defects.

The critical failure strain corresponding to the intrinsic strength ranged from 12% to 8% for the defective models with a zigzag edge interface and 14% to 9% for the defective models with an armchair edge interface, respectively. These values were comparable with the critical failure strain of the graphene/*h*-BN interface without defects and much lower than that of the pristine graphene or *h*-BN.

For the following discussion, we define the defect degree of vacancies as the linear density of the vacancies along the interface. It is desirable to reflect the influence of the defect degree of the interface on the strength of the graphene/*h*-BN sheet. So, the relationship between the intrinsic strength, the critical failure strain and the linear density of the vacancies along the interface was studied. With limited computing resource, just one type of vacancy defect was selected on each interface edge, *i.e.*, single carbon vacancy on the zigzag (C-B) edge interface and single nitrogen vacancy on the armchair edge interface. As shown in Fig. 3(d), due to the damage of the vacancies to the interface, the strength of both the zigzag interface and armchair interface decrease with the increasing of the defect degree. A similar trend was also observed between the critical failure strain and the defect degree. As shown in Fig. 2, the missing carbon (or nitrogen) atoms at the vacancy introduced dangling bonds around the vacancy defect on the interface, which break the integrity of the whole surface. Thus, increasing the defect degree had caused a more detrimental effect on the graphene/*h*-BN interface.

On the other hand, the relationship between the inflection angles and the defect density based on the analysis of these two defect types were discussed. The inflection angles were summarized in Table S2. The dependence of inflection angles on the linear density was shown in Figure S2. It was found that along with the increasing linear density of the defects, the inflection angle of the system basically increased to stabilize the graphene/h-BN structure. In addition, the increase of the inflection angle reduces the intrinsic strength of the graphene/h-BN sheet to a certain degree. This trend was in agreement with the relationship between the intrinsic strength and inflection angles.

### Failure Behavior of the ghraphene/*h*-BN interfaces

**Figure 6:**
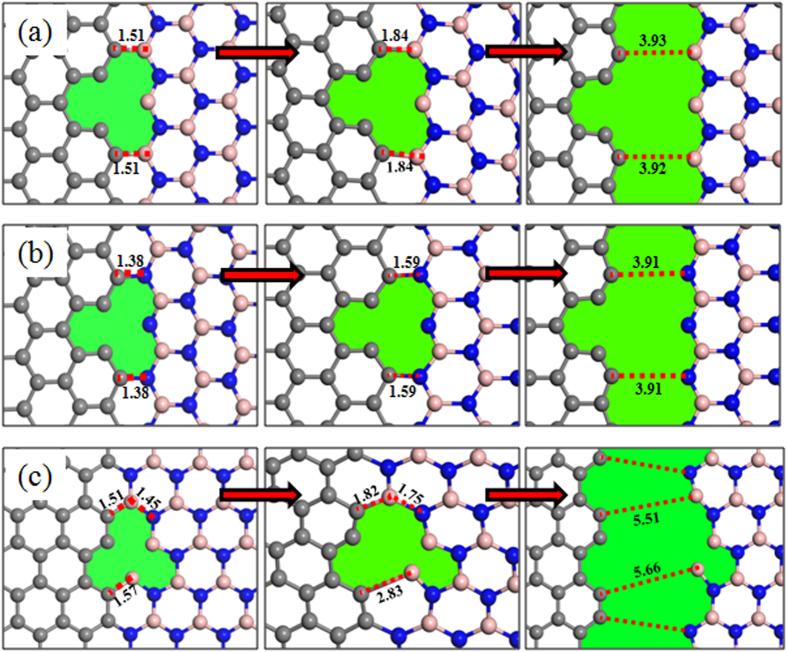
Fracture processes of the interface with (**a**) C vacancies at the C-B zigzag interface; (**b**) C vacancies at the C-N zigzag interface; (**c**) N vacancies at the armchair interface.

To reveal the fracture process of the graphene/*h*-BN interface due to the tensile load, the configuration of the interfaces with different strains were observed. The fracture process for several typical interfaces with defects was collected in Fig. 6. The fracture for all graphene/*h*-BN sheets occurred at or near the interface, which indicated that the interface had weak mechanical strength of such material. For a zigzag interface without defects, the fracture appeared on the C-B interface rather than the C-N interface, as a C-N bond possessed shorter bond length and higher interaction energy than a C-B bond. When the vacancies were located at the C-B interface, still the C-B interface fractured with the elongation of C-B bonds under tensile load (shown in Fig. 6(a)). However, while the vacancies appeared on the C-N interface, the sheet fractured due to broken the C-N bonds (see Fig. 6(b)). It demonstrated that defects could significantly influence the surface strength. As for the graphene/*h*-BN sheet with an armchair interface, the fracture of an am-SV-N system began with the elongation of two C-B bonds around the vacancy. As shown in Fig. 6(c), the initial lengths of these two bonds are 1.51 Å and 1.57 Å, respectively. Due to stretching of the surface, two B-N bonds which connected with the corresponding C-B bond were also elongated. However, the final fracture still occurred with the breaking of C-B bonds. After this, the elongated B-N bonds basically turn back to their original bond length.

### Tuning the electronic properties by applying mechanical stain

Pioneer researches have demonstrated that mechanical straining can alter the band gaps of graphene nano-ribbons significantly^36^,^37^,^38^. Here, we give a discussion on the electronic band structure of the graphene/*h*-BN systems and the changes of the electronic band structure during the tensile process. Four models were selected as probes: (i) the defect-free graphene/h-BN systems with the zigzag edge interface; (ii) the defect-free graphene/h-BN systems with armchair edge interface; (iii) the zz-SV(CB)-C system with a carbon vacancy defect at the graphene/h-BN (C-B) interferce; (iv) the am-SV-N system with a nitrogen vacancy defect at the graphene/h-BN interface. For each model, the electronic band structure of the system in the absence of mechanical strain and that with mechanical strain closed to its critical failure strain, were calculated and analyzed.

The energy band figures were collected in the Supplementary information (Figures S3 and S4). The dependence of the electronic band structure on mechanical strain was observed. For the defect-free graphene/h-BN interface with a zigzag edge interface, a semi-mental property could be identified clearly in the absence of strain. When 13% mechanical in-plane strain was applied perpendicular to the zigzag graphene/h-BN interface, energy band near the Fermi lever changed and the interface become metallic. For the defect-free graphene/h-BN interface with an armchair interface, the applied mechanical strain reduced the band gap of the system from 0.85 eV to 0.63 eV. This phenomenon was also observed in a pristine h-BN sheet^38^. As shown in Figure S4, the defective interface (zigzag edge) with a carbon vacancy will change from an insulator to a metal when applying mechanical strain on it. While for the defective interface (armchair edge) with a nitrogen vacancy, it will change from a metal to an insulator. These changes may be caused by the electron redistribution during the tensile process. The results indicate that mechanical straining is an effective way to tune the electrical properties of graphene/h-BN hybrid interface.

## Conclusions

In summary, twelve important typical vacancy defects of the hybrid graphene/*h*-BN sheet, which were located at the interface between graphene and *h*-BN domains, were studied by DFT method. The interface structure, mechanical properties, changes of the energy band during tensile process, and failure behavior of the hybrid graphene/*h*-BN sheet were investigated and compared. The results showed that the formation energy of the defective graphene/*h*-BN interface basically increased with increasing inflection angle. While Young’s modulus for all of the graphene/*h*-BN systems decreased with increasing inflection angle. The intrinsic strength of the hybrid graphene/*h*-BN sheets was affected not only by the inflection angles but also by the type of the interface connection and the type of the defects. The electronic properties of the hybrid interface might be affected significantly by the mechanical strain. The information obtained in this work would provide fundamental information for the application of the hybrid graphene/*h*-BN materials in nano-devices.

## Additional Information

**How to cite this article**: Ding, N. *et al*. Mechanical properties and failure behaviors of the interface of hybrid graphene/hexagonal boron nitride sheets. *Sci. Rep.* **6**, 31499; doi: 10.1038/srep31499 (2016).

## Supplementary Material

Supplementary Information

## Figures and Tables

**Table 1:** Parameters for the graphene/*h*-BN interface including the periodic length along the interface line *L,* inflection angle *α*, formation energy *E*_*f*_, Young’s modulus *E*, intrinsic strength *τ* and critical failure strain *δ*.

Systems	*L* (Å)	Inflection angle *α* (deg)	Formation energy *E*_*f*_ (eV)	Young’s modulus *E* (GPa)	Intrinsic strength *τ* (GPa)	Critical failure strain *δ* (%)
Graphene	4.3(zz)/2.5(am)#	0	/	1032.2*	120(zz)/100(am)**,#	19(zz)/23(am)**,#
*h*-BN	4.4(zz)/2.5(am)#	0	/	863(zz)/996(am)#	103(zz)/87(am)***,#	24(zz)/18(am)***,#
BN-G (zz edge)	2.5	10	/	573	84	14
BN-G (am edge)	4.3	3	/	744	95	17
zz-SV(CB)-C	7.4	8	7.75	555	55	10
zz-SV(CB)-B	7.4	17	8.36	541	48	8
zz-DV(CB)	7.4	30	14.44	228	62	9
zz-SV(CN)-C	7.4	19	8.18	479	76	12
zz-SV(CN)-N	7.4	18	6.32	570	64	12
zz-DV(CN)	7.4	20	13.05	499	74	11
am-SV-N	8.5	15	7.01	623	75	13
am-SV-B	8.5	17	8.05	616	58	9
am-SV-C(N)	8.5	15	8.17	663	65	11
am-SV-C(B)	8.5	5	7.62	700	58	14
am-DV(BN)	8.5	30	10.69	503	33	10
am-DV(CC)	8.5	35	11.58	303	47	14

^*^Ref. 35.

^**^Ref. 4.

^***^Ref. 5.

^#^(zz)/(am) labeled for the pristine graphene or *h*-BN means the load direction (zz for zigzag direction; am for armchair direction), rather than the interface edge shape.

## References

[b1] Grantab R., Shenoy V. B. & Ruoff R. S. Anomalous strength characteristics of tilt grain boundaries in graphene. Science 330, 946–948 (2010). 21071664 10.1126/science.1196893

[b2] Geim A. K. & Novoselov K. S. The rise of graphene. Nature Mater. 6, 183–191 (2007). 17330084 10.1038/nmat1849

[b3] Lee C., Wei X. D., Kysar J. W. & Hone J. Measurement of the elastic properties and intrinsic strength of monolayer graphene. Science 321, 385–388 (2008). 18635798 10.1126/science.1157996

[b4] Zhang J. F., Zhao J. J. & Lu J. P. Intrinsic strength and failure behaviors of graphene grain boundaries. ACS Nano 6, 2704–2711 (2012). 22369492 10.1021/nn3001356

[b5] Ding N., Wu C.-M. L. & Li H. Effect of grain boundaries on the mechanical properties and failure behavior of hexagonal boron nitride sheets. Phys. Chem. Chem. Phys. 16, 23716–23722 (2014). 25271794 10.1039/c4cp02521k

[b6] Wei Y. J. . The nature of strength enhancement and weakening by pentagon-heptagon defects in graphene. Nature Mater. 11, 759–763 (2012). 22751178 10.1038/nmat3370

[b7] Han T. W., Luo Y. & Wang C. Y. Effects of temperature and strain rate on the mechanical properties of hexagonal boron nitride nanosheets. J. Phys. D: Appl. Phys. 47, 025303 (2014).

[b8] Berdiyorov G. R. & Peeters F. M. Influence of vacancy defects on the thermal stability of silicene: a reactive molecular dynamics study. RSC Advances 4, 1133–1137 (2014).

[b9] Jhon Y. I., Zhu S. E., Ahn J. H. & Jhon M. S. The mechanical responses of tilted and non-tilted grain boundaries in graphene. Carbon 50, 3708–3716 (2012).

[b10] Jhon Y. I. . Grain boundaries orientation effects on tensile mechanics of polycrystalline graphene. RSC Advances 3, 9897–9903 (2013).

[b11] Ci L. J. . Atomic layers of hybridized boron nitride and graphene domains. Nat. Mater. 9, 430–435 (2010). 20190771 10.1038/nmat2711

[b12] Levendorf M. P. . Graphene and boron nitride lateral heterostructures for atomically thin circuitry. Nature 488, 627–632 (2012). 22932386 10.1038/nature11408

[b13] Gao Y. B. . Toward single-layer uniform hexagonal boron nitride- graphene path works with zigzag linking edges. Nano Lett. 13, 3439–3443 (2013). 23758663 10.1021/nl4021123

[b14] Ramasubramaniam A. & Naveh D. Carrier-induced antiferromagnet of graphene islands embedded in hexagonal boron nitride. Phys. Rev. B 84, 075405 (2011).

[b15] Jiang J. W., Wang J. S. & Wang B. S. Minimum thermal conductance in graphene and boron nitride superlattice. Appl. Phys. Lett. 99, 043109 (2011).

[b16] Son Y. W., Cohen M. L. & Louie S. G. Half-metallic Graphene Nanoribbons. Nature 444, 347–349 (2006). 17108960 10.1038/nature05180

[b17] Bhowmick S., Singh A. K. & Yakobson B. I. Quantum dots and nanoroads of graphene embedded in hexagonal boron nitride. J. Phys. Chem. C 115, 9889–9893 (2011).

[b18] Dean C. R. . Boron nitride substrates for high-quality graphene electronics. Nat.Nanotechnol. 5, 722–726 (2010). 20729834 10.1038/nnano.2010.172

[b19] Gann ett W. . Boron nitride substrates for high mobility chemical vapor deposited graphene. Appl. Phys. Lett. 98, 242105 (2011).

[b20] Lee K. H. . Large-scale synthesis of high-quality hexagonal boron nitride nanosheets for large-area graphene electronics. Nano Lett. 12, 714–718 (2012). 22220633 10.1021/nl203635v

[b21] Zomer P. J., Dash S. P., Tombros N. & van, Wees B. J. A transfer technique for high mobility graphene devices on commercially available hexagonal boron nitride. Appl. Phys. Lett. 99, 232104 (2011).

[b22] Nakamura J., Nitta T. & Natori A. Electronic and magnetic properties of BNC ribbons. Phys. Rev. B 72, 205429 (2005).

[b23] Ding N. . Structures and electronic properties of vacancies at the interface of hybrid graphene/hexagonal boron nitride sheet. Comput. Mater. Sic. 117, 172–179 (2016).

[b24] Fan Y. C. . Manifold electronic structure transition of BNC biribbons. J. Appl. Phys. 110, 034314 (2011).

[b25] Fan Y. C. . Theoretical insights into the built-in electric field and band offsets of BN/C heterostructured zigzag nanotubes. 44, 095405 (2011).

[b26] Lu J., Gomes L. C., Nunes R. W., Castro Neto A. H. & Loh K. P. Lattice relaxation at the interface of two-dimentional crystals: graphene and hexagonal boron-nitride. Nano Lett. 14, 5133–5139 (2014). 25083603 10.1021/nl501900x

[b27] Thomas T., Latham C. D., Heggie M. I., Briddon P. R. & Rayson M. J. Vacancy diffusion and coalescence in graphene directed by defect strain fields. Nanoscale 6, 2978–2986 (2014). 24487384 10.1039/c3nr06222h

[b28] Güryel S. . Effect of structural defects and chemical functionalisation on the intrinsic mechanical properties of graphene. Phys. Chem. Chem. phys. 15, 659–665 (2013). 23187874 10.1039/c2cp43033a

[b29] Chavez-Castillo M. R., Rodriguez-Meza M. A. & Meza-Montes L. Size, vacancy and temperature effects on Young’s modulus of silicene nanoribbons. RSC Advances 5, doi: 10.1039/C5RA15312C (2015). PMC9078962 35541532

[b30] Liu Z. . In-plane heterostructures of graphene and hexagonal boron nitride with controlled domain sizes. Nature nanotechnology 8, 119–124 (2013). 10.1038/nnano.2012.256 23353677

[b31] Drost R. . Electronic states at the graphene-hexagonal boron nitride zigzag interface. Nano Lett. 14, 5128–51132 (2014). 25078791 10.1021/nl501895h

[b32] Zhang M. Y., Li G. S. & Li L. P. Graphene nanoribbons generate a strong third-order nonlinear optical response upon intercalating hexagonal boron nitride. J. Mater. Chem. C 2, 1482 (2014).

[b33] Yang K. . Enhanced thermoelectric properties in hybrid graphene/boron nitride nanoribbons. Phys. Rev. B 86, 045425 (2012).

[b34] Perdew J. P., Burke K. & Ernzerhof M. Generalized Gradient Approximation Made Simple. Phys. Rev. Lett. 77, 3865–3868 (1996). 10062328 10.1103/PhysRevLett.77.3865

[b35] Jing N. N., Xue Q. Z. & Ling C. C. Effect of defects on Young’s modulus of graphene sheets: a molecular dynamics simulation. RSC Advances 2, 9124–9129 (2012).

[b36] Han M., Zyilmaz B., Zhang Y. & Kim P. Energy band-gap engineering of graphene nanoribbons. Phys. Rev. Lett. 98, 206805 (2007). 17677729 10.1103/PhysRevLett.98.206805

[b37] Machado-Charry E., Boulanger P., Genovese L., Mousseau N. & Pochet P. Tunable magnetic states in hexagonal born nitride sheets. Appl. Phys. Lett. 101, 132405 (2012).

[b38] Wu J. T., Wang B. L., Wei Y. J., Yang R. G. & Dresselhaus M. Mechanics and mechanically tunable band gap in single-layer hexagonal boron-nitride. Mater. Res. Lett. 1, 200–206 (2013).

