# Supplementary material for: Mechanical properties and failure behaviors of the interface of hybrid graphene/hexagonal boron nitride sheets
Source: Sci Rep. 2016 Aug 16;6:31499. doi: 10.1038/srep31499 (PMC4985750; doi:10.1038/srep31499)
Supplement: Supplementary Information [file srep31499-s1.doc]

**Mechanical properties and failure behaviors of the interface of hybrid graphene/hexagonal boron nitride sheets**

**Supplementary information**

Ning Ding **^a,b^*, Xiangfeng Chen*^b^*, Chi-Man Lawrence Wu**^a,b^*

*^a^* Department of Physics and Materials Science, City University of Hong Kong,

Hong Kong SAR, PR China

*^b^* Shandong Academy of Sciences, Jinan, PR China

*Address correspondence to: Prof. Chi-Man Lawrence Wu, Department of Physics and Materials Science, City University of Hong Kong, Hong Kong SAR, PR China. E-mail: lawrence.wu@cityu.edu.hk (C.-M.L. Wu)

Dr. Ning Ding, Shandong Academy of Sciences, Jinan, PR China. E-mail: dingningch@aliyun.com (N. Ding)

**Figure S1** Side view of a graphene/*h*-BN model. The inflection angle α was defined to denote the flection degree of the graphene/*h*-BN sheet. In details, it was defined as the angle between the graphene and *h*-BN domains.


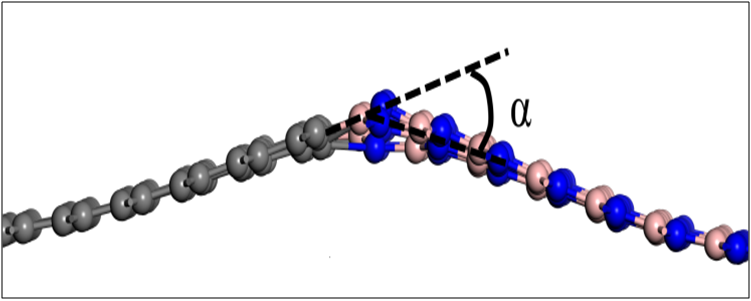


**Figure S2** Inflection angle as a function of the linear density of the graphene/*h*-BN systems with carbon vacancy defects for the zigzag edge and nitrogen vacancy defects for the armchair edge.


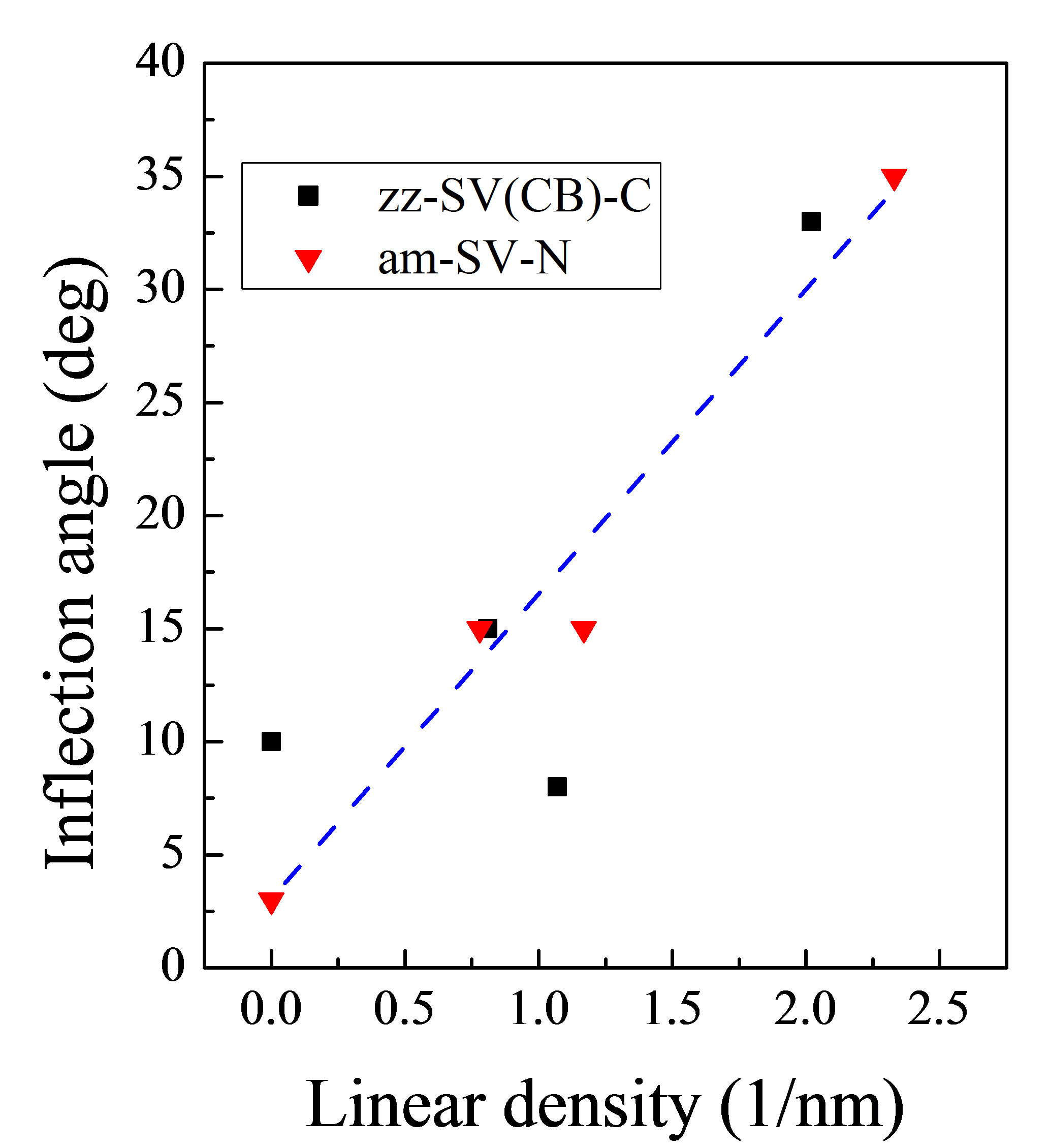


**Figure S3** Energy band for the systems of (a) defect-free BN-G (zz edge) with 0 % atomic strain; (b) defect-free BN-G (zz edge) with 13 % atomic strain; (c) defect-free BN-G (am edge) with 0% atomic strain; and (d) defect-free BN-G (am edge) with 16 % atomic strain. The Fermi level is set to be zero.


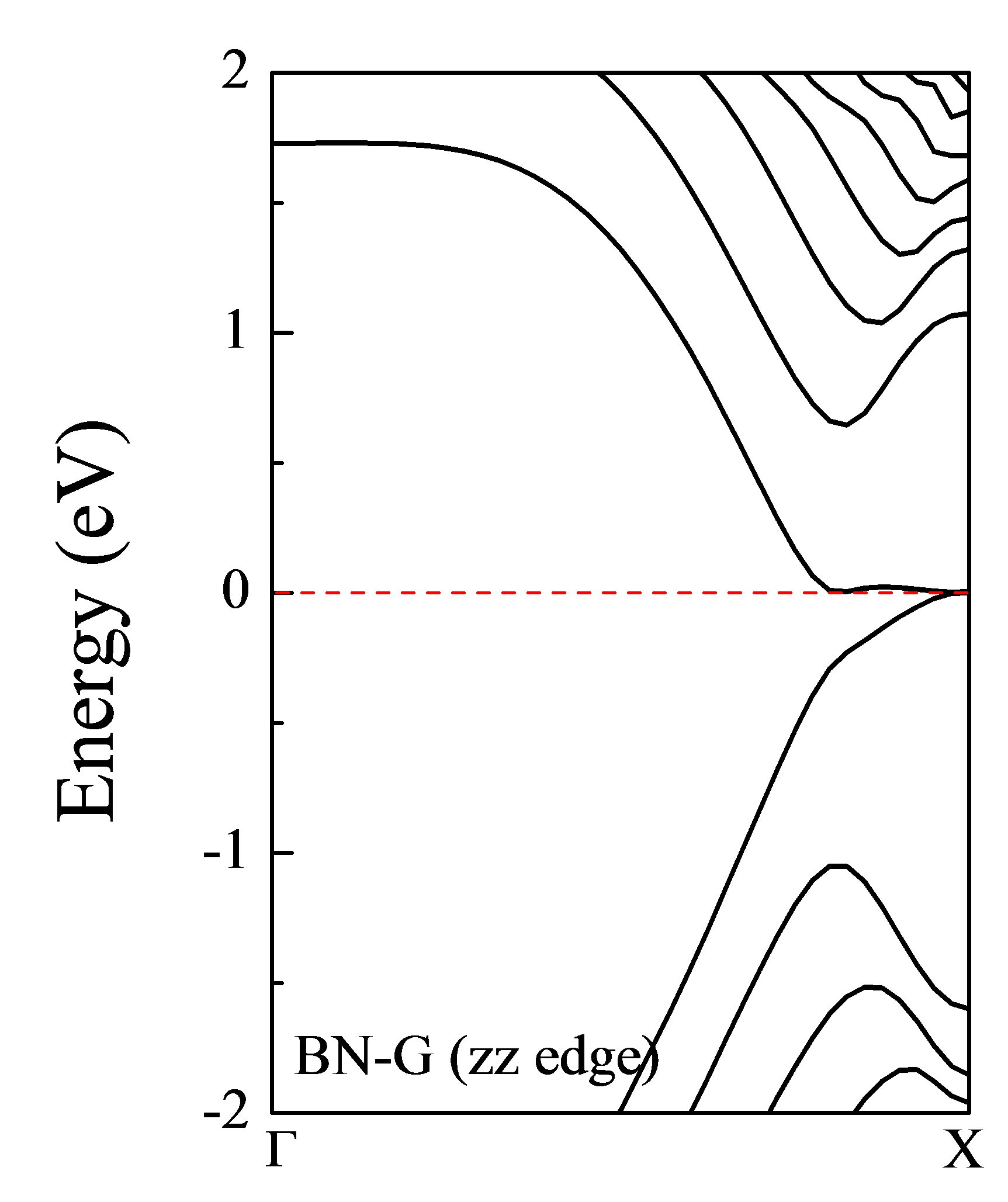

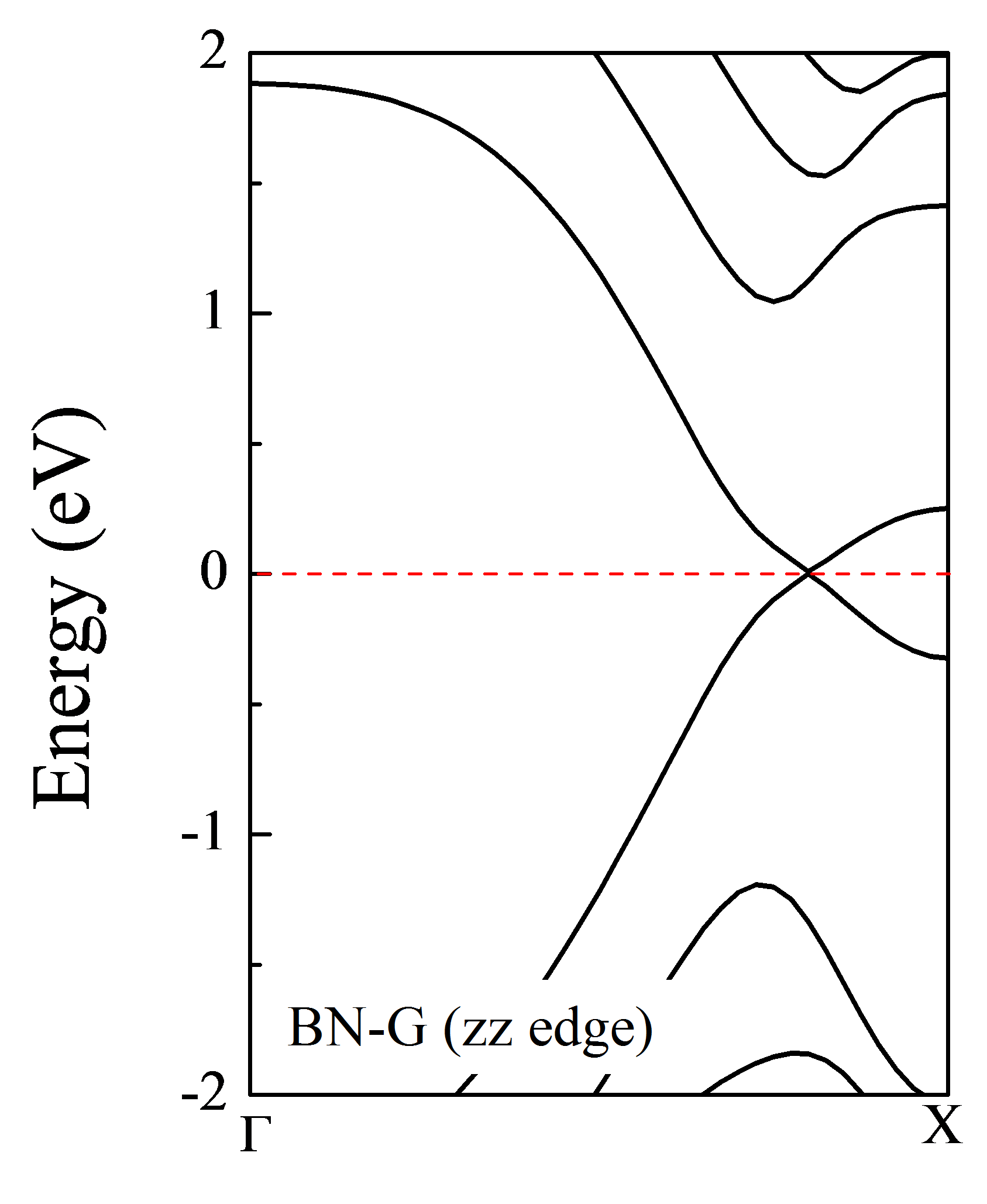


(a)

(b)

*δ=*0 % *δ=*13 %


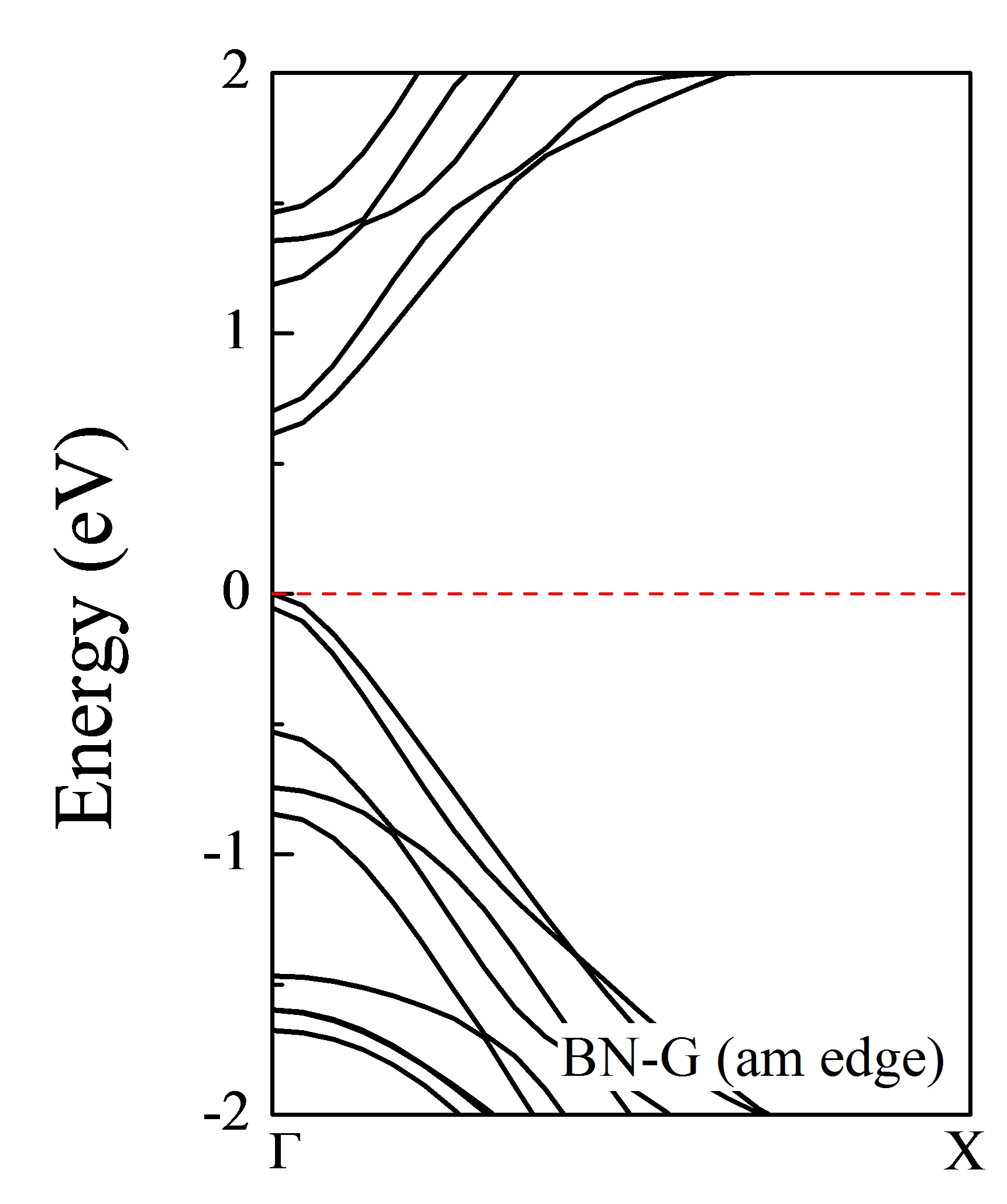

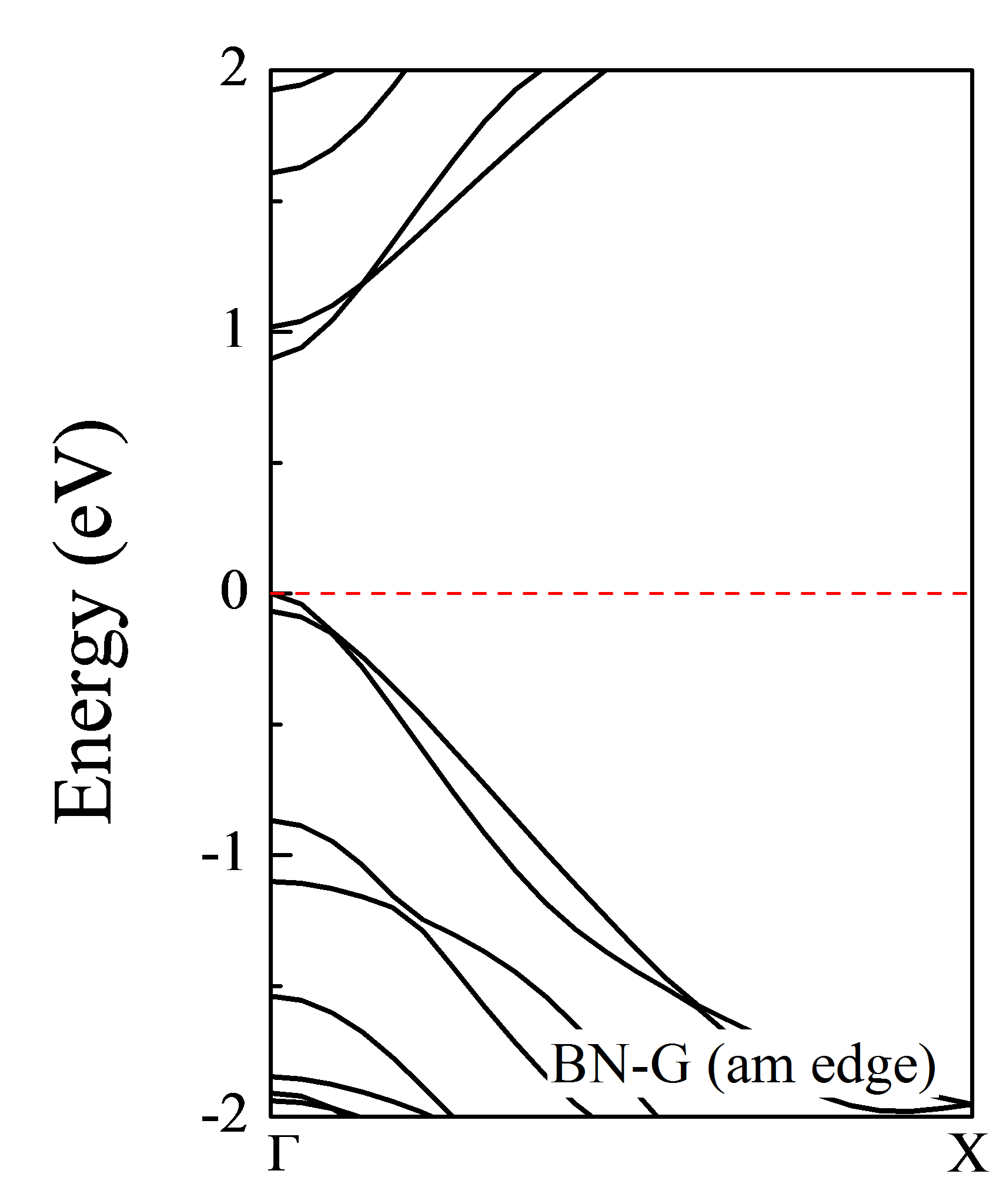


(c)

(d)

*δ=*0 % *δ=*16 %

**Figure S4** Energy band for the systems of (a) zz-SV(CB)-C with 0 % atomic strain; (b) zz-SV(CB)-C with 13 % atomic strain; (c) am-SV-N with 0% atomic strain; and (d) am-SV-N with 11 % atomic strain. The Fermi level is set to be zero.

**
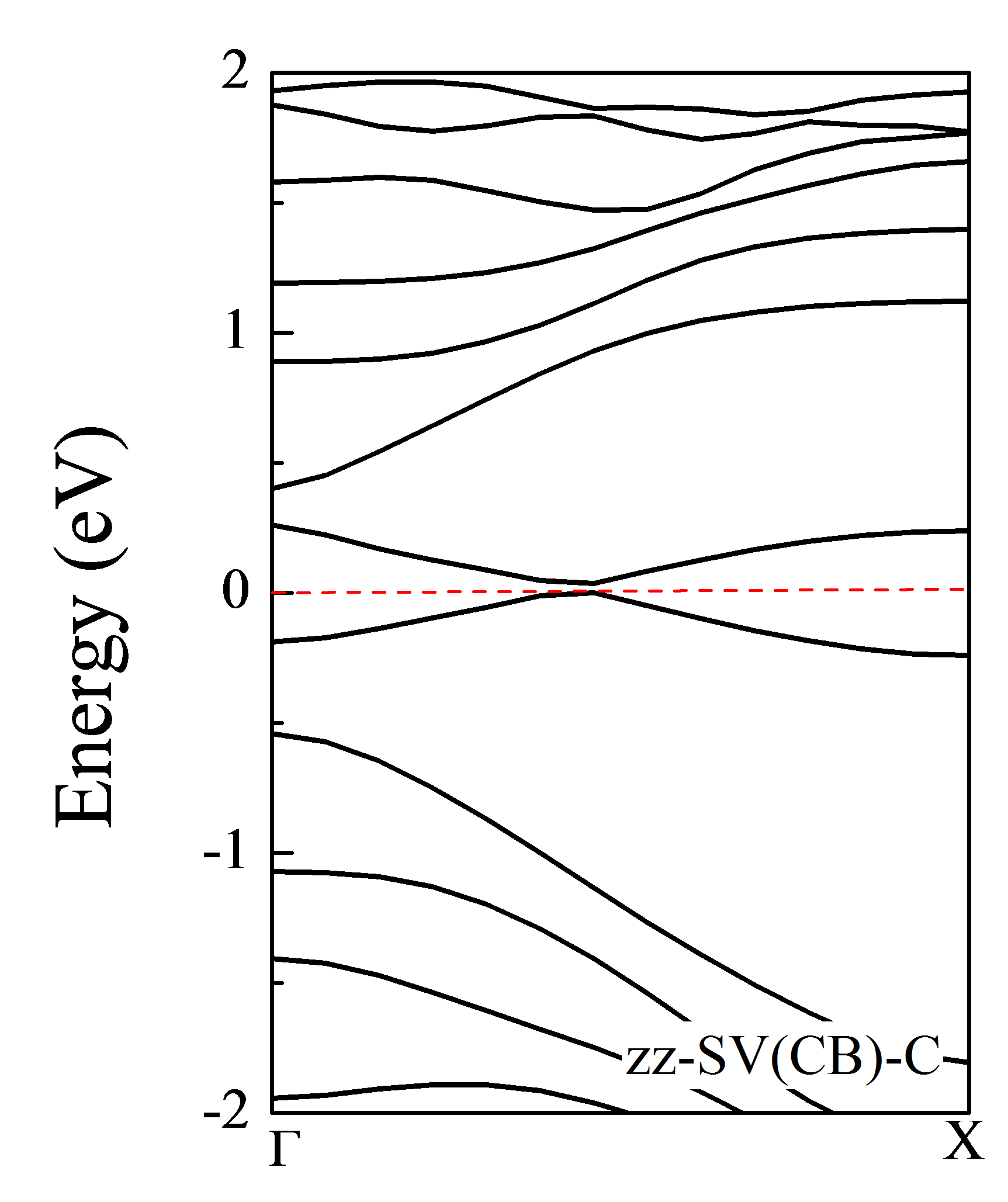

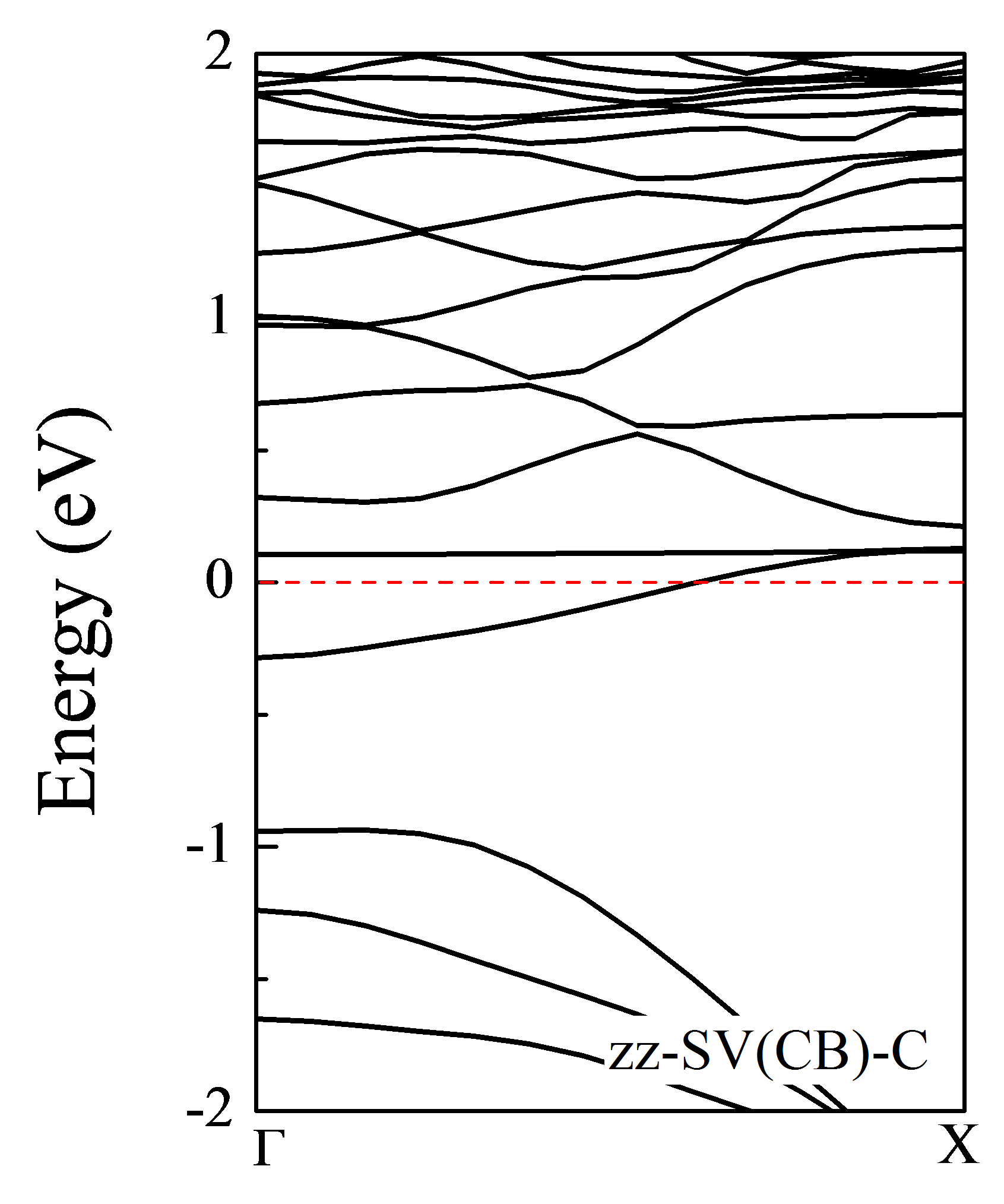
**

(a)

(b)

*δ=*0 % *δ=*9%

**
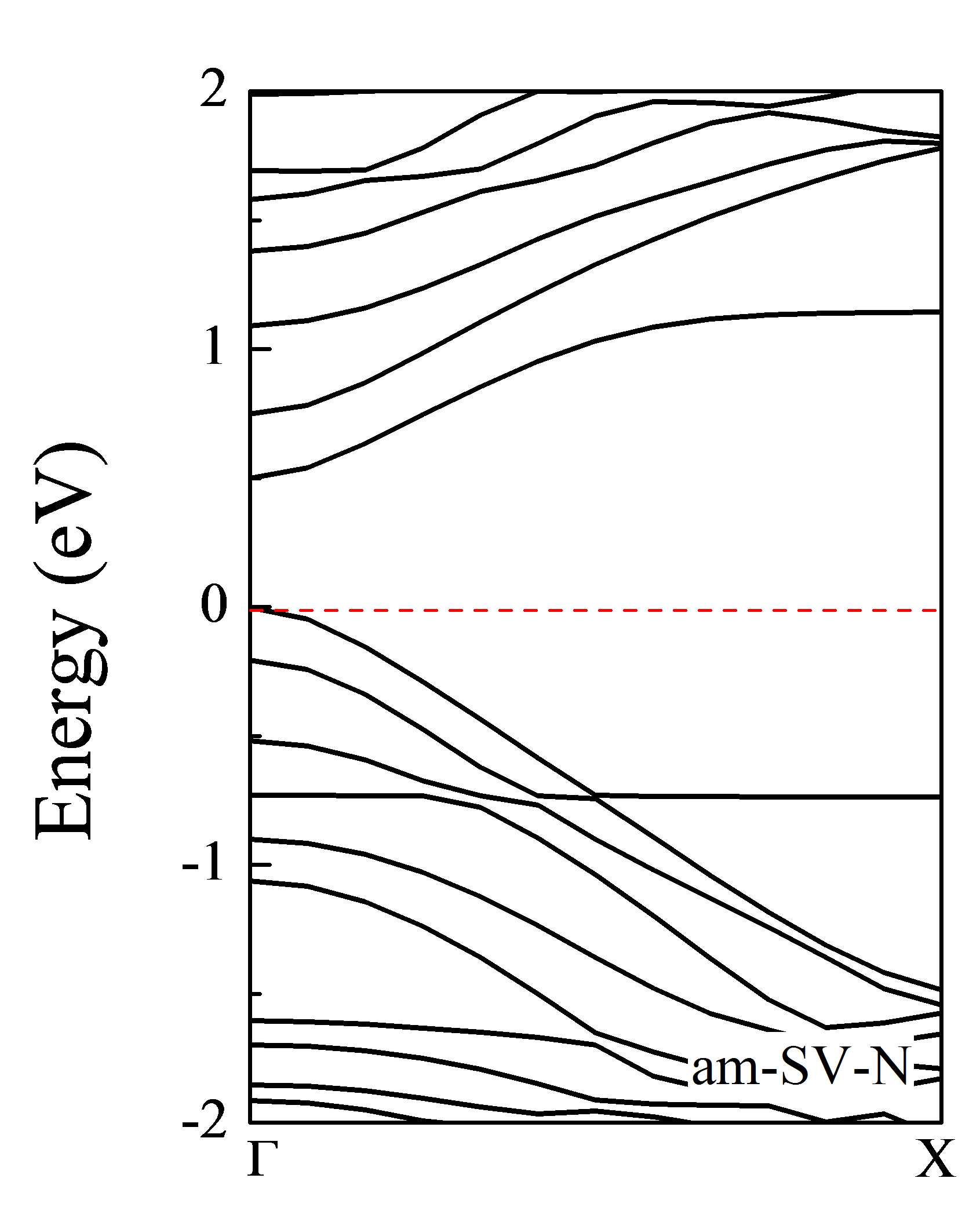

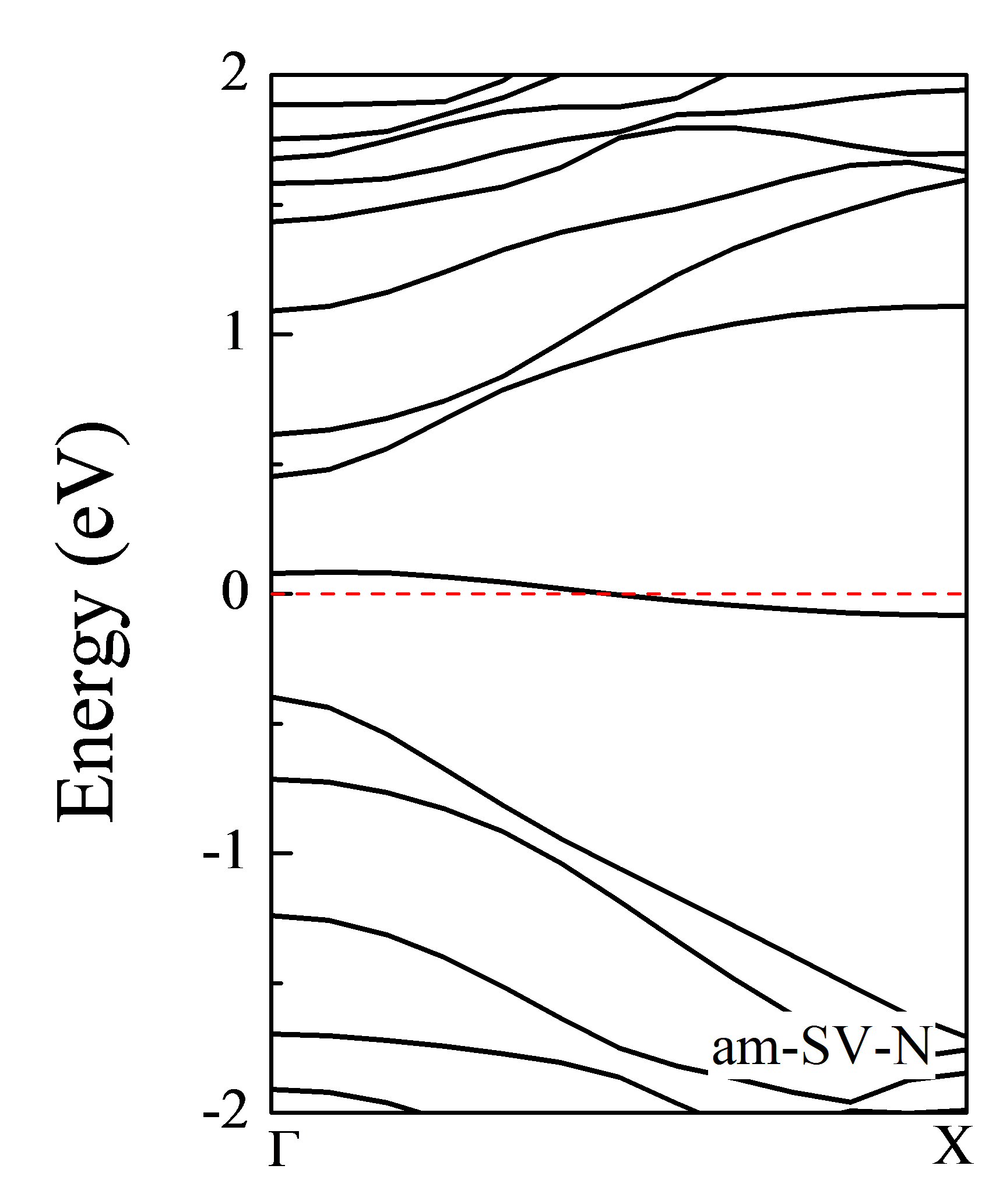
**

(c)

(d)

*δ=*0 % *δ=*11%

**Table S1** Detailed structural parameters around the vacancy including bond lengths, angles and atomic elevations.

| Systems | Bond length (Å) | | | | Angles(deg) | | | | | | | Elevation ( Å) | | |
| --- | --- | --- | --- | --- | --- | --- | --- | --- | --- | --- | --- | --- | --- | --- |
|  | C-B | B-N | C-C | C-N | C-C-B | C-C-C | C-B-N | N-B-N | B-N-B | C-N-B | C-C-N | B | N | C |
| Graphene | / | / | 1.42 | / | / | 120 | / | / | / | / | / | / | / | / |
| *h*-BN | / | 1.44 | / | / | / | / | / | 120 | 120 | / | / | / | / | / |
| BN-G (zz edge) | 1.56 | 1.44～1.46 | 1.42 | 1.41 | 119 | 120～122 | 121 | 119 | 119 | 121 | 119 | / | / | / |
| BN-G (am edge) | 1.50 | 1.43～1.45 | 1.40～1.41 | 1.40 | 121 | 120～122 | 120 | 120～121 | 119 | 122 | 120 | / | / | / |
| zz-SV(CB)-C | 1.51 | 1.44～1.46 | 1.38～1.44 | / | 115～118 | 119～126 | 111～120 | / | 113～125 | / | / | 0.45 | / | / |
| zz-SV(CB)-B | 1.50～1.56 | 1.41～1.56 | 1.40～1.50 | / | 107～122 | 114～128 | 112～121 | 120～130 | 116 | / | / | / | 0.54 | / |
| zz-DV(CB) | 1.57 | 1.41～1.42 | 1.40～1.43 | / | 122 | 114～118 | 125 | 122 | 112 | / | / | / | / | / |
| zz-SV(CN)-C | / | 1.39～1.49 | 1.39～1.44 | 1.38 | / | 117～119 | / | 121 | / | 120 | 124 | / | 0.58 | / |
| zz-SV(CN)-N | / | 1.42～1.53 | 1.40～1.50 | 1.37～1.39 | / | 115～125 | / | 115 | 118～121 | 112～119 | 111～118 | / | 0.35 | / |
| zz-DV(CN) | / | 1.43～1.52 | 1.40～1.44 | 1.41 | / | 115～117 | / | 114 | 115 | 120 | 122 | / | / | / |
| am-SV-N | 1.50～1.57 | 1.43～1.47 | 1.38～1.43 | / | 120～122 | 117～122 | / | 116 | 117～122 | / | / | / | / | / |
| am-SV-B | 1.30～1.35 | 1.40～1.44 | 1.41～1.58 | / | / | 126 | / | 112～127 | 115 | 115 | 116～117 | / | / | / |
| am-SV-C(N) | 1.54～1.61 | 1.40～1.46 | 1.41～1.56 | / | 116 | 111～128 | 124～126 | 123 | 116 | / | / | / | 0.58 | / |
| am-SV-C(B) | / | 1.43～1.70 | 1.40～1.45 | 1.36 | / | 114～123 | / | 115 | 112 | 112 | 111 | / | / | / |
| am-DV(BN) | 1.49 | 1.43～1.50 | 1.40～1.46 | 1.38 | 113 | 123 | 111 | 124 | 106～120 | 106 | 112 | / | / | / |
| am-DV(CC) | 1.49 | 1.45～1.49 | 1.41～1.46 | 1.38 | 104 | 113～125 | 110 | 124 | 120 | 108 | 111 | / | / | / |

**Table S2** Parameters for the graphene/*h*-BN systems with different defect linear densities (carbon vacancy defects for the zigzag edge and nitrogen vacancy defects for the armchair edge), including the linear density, inflection angle, intrinsic strength and critical failure strain.

| Systems | Linear density  (1/nm) | Inflection angle  (deg) | Intrinsic strength  (GPa) | Critical failure strain  (%) |
| --- | --- | --- | --- | --- |
| zz-SV(CB)-C1 | 0 | 10 | 84 | 14 |
| zz-SV(CB)-C2 | 0.81 | 15 | 60 | 13 |
| zz-SV(CB)-C3 | 1.07 | 8 | 55 | 10 |
| zz-SV(CB)-C4 | 2.02 | 33 | 42 | 8 |
| am-SV-N1 | 0 | 3 | 95 | 17 |
| am-SV-N2 | 0.78 | 15 | 82 | 16 |
| am-SV-N3 | 1.17 | 15 | 75 | 13 |
| am-SV-N4 | 2.33 | 35 | 37 | 7 |
